# Manipulating transient SOT-MRAM switching dynamics for efficiency improvement and probabilistic switching

**DOI:** 10.1038/s41598-025-22014-1

**Published:** 2025-10-31

**Authors:** Shreyes Nallan, Jian-Gang Zhu

**Affiliations:** Data Storage Systems Center, Department of Electrical and Computer Engineering, Carnegie Mellon University, Pittsburgh, PA 15213 USA

**Keywords:** Electrical and electronic engineering, Magnetic devices, Electronic and spintronic devices

## Abstract

In this paper, we investigate the effect of transient dynamics in the switching process for spin-orbit torque magnetic random-access memory (SOT-MRAM) devices stabilized by in-plane uniaxial magnetocrystalline anisotropy. We develop theory for the interaction between spin torques and effective fields during a magnetization write trajectory and apply this framework to find regions of failed and successful switching. We focus particularly on a “quasi-stochastic” regime located between regions of deterministic failed and successful switching and caused by the interplay between torque-driven and precession-driven magnetization evolution during the switching process. We demonstrate a series of minor alterations to device geometry, material characteristics, and electrical inputs that use transient phenomena to lower the switching barrier—thereby allowing for SOT-MRAM switching with significantly lower currents and faster write speeds than the traditional architecture. Furthermore, we demonstrate that at elevated temperatures, the unpredictable stochastic regime evolves into a probabilistic “transition band” with clearly defined, montotonic, and tunable regions of probabilistic operation. Through this addition of control mechanisms through electrical inputs, our framework paves the way for the creation of a fast, efficient probabilistic bit (p-bit) for the field of probabilistic computing.

## Introduction

Recently, spin-orbit torque magnetic random-access memory (SOT-MRAM) devices have gained prominence in the field of memory recording for their speed, efficiency, and potential for spintronic integration^1–3^. In this device framework, magnetization states can be written onto individual cells through direct application of electrical currents, removing the need for electromagnetic write heads that require mechanical actuation and operate through inefficient and slow stray fields^4–6^. In addition, the SOT-MRAM framework directs the flow of current through a metallic underlayer and away from the magnetic cells themselves, ensuring the stability and longevity of the magnetic materials^7,8^. Despite these benefits, however, SOT-MRAM devices are subject to fundamental scalability challenges due to the external fields^1,9^ or elliptical aspect ratios^10,11^ needed to provide the required energetic stabilization^12^. In previous work, we presented a new architecture that overcomes these issues: an SOT-MRAM device stabilized by in-plane magnetocrystalline anisotropy^13^.

Our previous simulations showed that SOT-MRAM devices exhibit complex and unexpected transient behavior, including a quasi-stochastic regime between regions of deterministic failed and successful switching^13,14^. However, the exact reasons for these phenomena remained obscure, and without this knowledge, transient behavior remained an unpredictable impediment to desired switching behavior.

In this work, we trace the origin of transient dynamics in the SOT-MRAM switching process to the interaction between the spin-injection-induced transfer of angular momentum and the natural precession of magnetization under the influence of anisotropy and demagnetization fields. In the “Transient dynamics in in-plane SOT-MRAM” section , we develop a theory to describe and help manipulate this vital interaction, focusing on the counterveiling effects of static energetic barriers and time-dependent precessional and spin-torque-driven motion. In the “Improving performance through zero-temperature effects” section, we use this fundamental knowledge to our advantage. Based on transient simulations, we propose a set of alterations in device architecture, material properties, and/or electrical inputs to help reduce the critical current needed to switch state in SOT-MRAM devices while keeping all other parameters constant. We thereby open the door to significant improvements in memory efficiency and speed with only minor changes to the well-known SOT-MRAM architecture. Finally, in the “Probabilistic switching through thermal effects” section, we introduce thermal fluctuations into our system and demonstrate the possibility of probabilistic switching with well-defined and tunable bands of switching probability, coupled with a complete alleviation of the zero-temperature stochastic regime. In this way, we create a precisely controllable probabilistic bit (p-bit) for use in the increasingly prominent field of probabilistic computing^15,16^. The p-bit derived from these investigations displays faster speed, better efficiency, and finer control over weight and stability than any found in the current literature. These discoveries pave the way for faster and more prevalent probabilistic computers based on SOT-MRAM technology.

## Transient dynamics in in-plane SOT-MRAM

**Figure 1:**
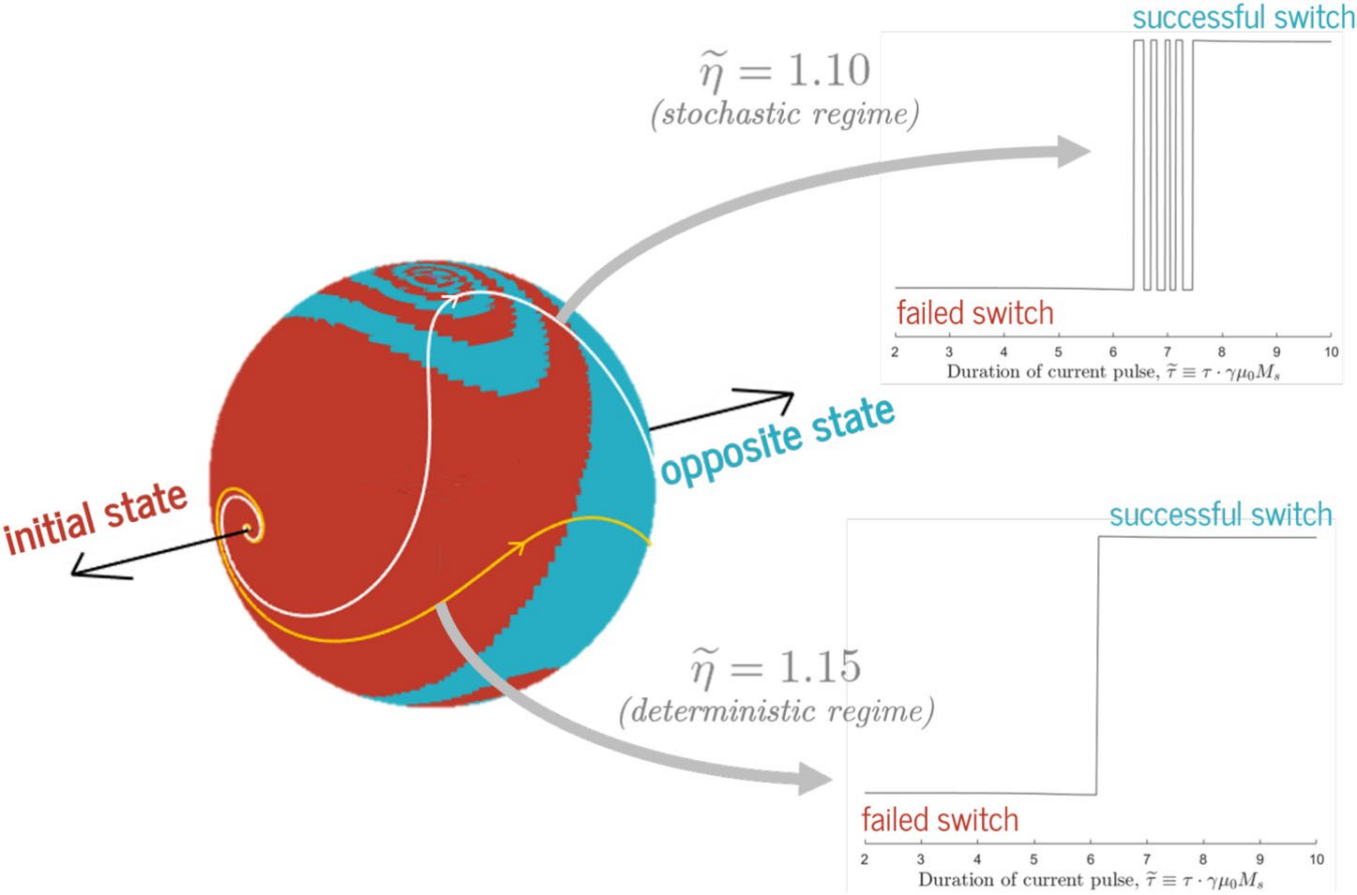
In our devices, an $\hat{m}$ starting from the red regions will fall back to the initial state; an $\hat{m}$ starting from the blue regions will precess to the final state. A spin pulse with magnitude $\eta$ and angle $\beta$ will send $\hat{m}$ along a preset trajectory; two example trajectories are shown in white and yellow. Stopping the pulse when $\hat{m}$ has reached a red region leads to failed switching and stopping it in a blue region leads to successful switching. This creates the complex switching behaviors shown on the right: some trajectories create deterministic transitions between failed and successful switching, while others create intricate quasi-stochastic behavior.

### Simulation setup and methodology

In previous work^13,14^, we discussed our macrospin model for the magnetization trajectory of a memory cell stabilized by in-plane uniaxial magnetocrystalline anisotropy and switched by a spin-transfer-torque pulse originating from an underlayer charge current. There are five independent factors affecting the evolution of the magnetic state: (1) the strength of the uniaxial anisotropy, *K*; (2) the direction of the in-plane easy axis, $\hat{k}$; (3) the magnitude of the input spin pulse, $\eta$; (4) the duration or pulse width of the input spin pulse, $\tau$; and (5) the injected spin vector, $\hat{p}$, from which we derive a polarization angle, $\beta$, defined as the in-plane angle between $\hat{p}$ and $\hat{k}$.

The time dynamics of the magnetization $\hat{m}$ are found through the explicit Landau-Lifshitz-Gilbert equation with Slonczewski’s modification for spin transfer torque^17^:

1 $$\begin{aligned} \dfrac{d\hat{m}}{d\widetilde{t}} = -\frac{1}{1+\alpha ^2}\left( \hat{m} \times \left[ \widetilde{\textbf{B}} + \alpha \hat{m} \times \widetilde{\textbf{B}} - \alpha \widetilde{\eta }\hat{p} - \widetilde{\eta } \hat{p}\times \hat{m}\right] \right) \end{aligned}$$

where we have nondimensionalized all parameters using $\widetilde{\textbf{B}} \equiv \vec {B}/\mu _0M_s$, $\widetilde{\eta } \equiv \eta / \gamma \mu _0 M_s$, and $\widetilde{t} \equiv t \cdot \gamma \mu _0M_s$. The nondimensionalized effective field $\widetilde{\textbf{B}}$ changes over the course of the switching process and is the main driver of $\hat{m}$’s movement (especially after the spin pulse has expired). It comprises an anisotropy term $\widetilde{\textbf{B}}_{\text {ani}} \equiv \frac{2K}{\mu _0{M_s}^2}(\hat{m} \cdot \hat{k})\hat{k}$ and a demagnetization term $\widetilde{\textbf{B}}_{\text {demag}} \equiv -(\hat{m} \cdot \hat{z})\hat{z}$. At elevated temperature, it also includes a randomized thermal component, which will be covered in greater detail in “Probabilistic switching through thermal effects” section.

All analyses below are based on numerical simulations of this system of ordinary differential equations, which is generally intractable analytically due to its time-dependent and non-linear terms. With certain assumptions and simplifications, however, we can arrive at theoretical relationships that qualitatively confirm the functional forms of the numerical simulation results. The corresponding theoretical analysis can be found in the supplementary material for this manuscript.

### Switching regions

To understand the switching process described above, we must answer two questions:

1. When the current is on, what is the path of $\hat{m}$, caused mostly by torque from $\hat{p}$ in the influence of the effective fields?
2. After the current turns off, which final state does $\hat{m}$ damp to, influenced solely by the anisotropy and demagnetization fields?

The latter question is easier to answer, as it depends only on the nondimensionalized anisotropy constant $\widetilde{K} \equiv K / \mu _0{M_s}^2$. In Fig. 1, we point magnetizations along all points on a three-dimensional sphere and watch how they evolve. We color the regions of $\hat{m}$ that damp to the initial state and those that damp to the diametrically opposite state red and blue, respectively. We find that for magnetizations close to the plane of the film or either equilibrium state, this damping process works as expected: $\hat{m}$ goes to the closest easy-axis state. However, for magnetizations with strong demagnetizing-field contributions—those pointed out of the plane of the film or close to the borderline between the two easy-axis states—we find a fascinating whirl-like pattern caused by precession around the effective demagnetization field. In some cases, magnetizations that start off in the $+\hat{k}$ side of the sphere get flung to the $-\hat{k}$ final state (and vice versa).

We now add in the effect of the spin pulse. The injected spin torque causes $\hat{m}$ to follow a unique path, dependent on the magnitude $\eta$ and polarization vector $\hat{p}$ (corresponding to a polarization angle $\beta$). An example of such a trajectory is shown in white on Fig. 1. When the current switches off (i.e., when $t > \tau$), the magnetization stops evolving along the preset path and instead precesses around the effective fields. If we stop applying current while we are in the red region, the magnet will eventually fail to switch; if we stop the current while we are in the blue region, the magnet will succeed in switching. Sweeping the pulse duration $\tau$, which is equivalent to sweeping along the white path, produces a switching characteristic as shown in the bottom right of Fig. 1.

As seen in that figure, for any given $\eta$ and $\beta$, there is a wide initial range of $\tau$ for which switching will always fail and a wide final range for which switching will always succeed. These lower and upper bounds delineate regions of “safe”, predictable switching behavior, and their exact values are of great importance in device design. We have covered trends and implications of these parameters at length in previous work^13^.

The region in between these bounds corresponds with the passage of the magnetization trajectory through the whirl-type precession-based regime. We call it the “chaotic” or “stochastic regime”—not because there is any inherent nondeterminism in the governing equations but because the boundaries between failed and successful switching in this region are often so close together that no possible experiment can reasonably control the final state of $\hat{m}$. (These stochastic phenomena are not to be confused with the probabilistic switching behavior caused by the addition of random thermal noise, as covered in the “Probabilistic switching through thermal effects” section.)

**Figure 2:**
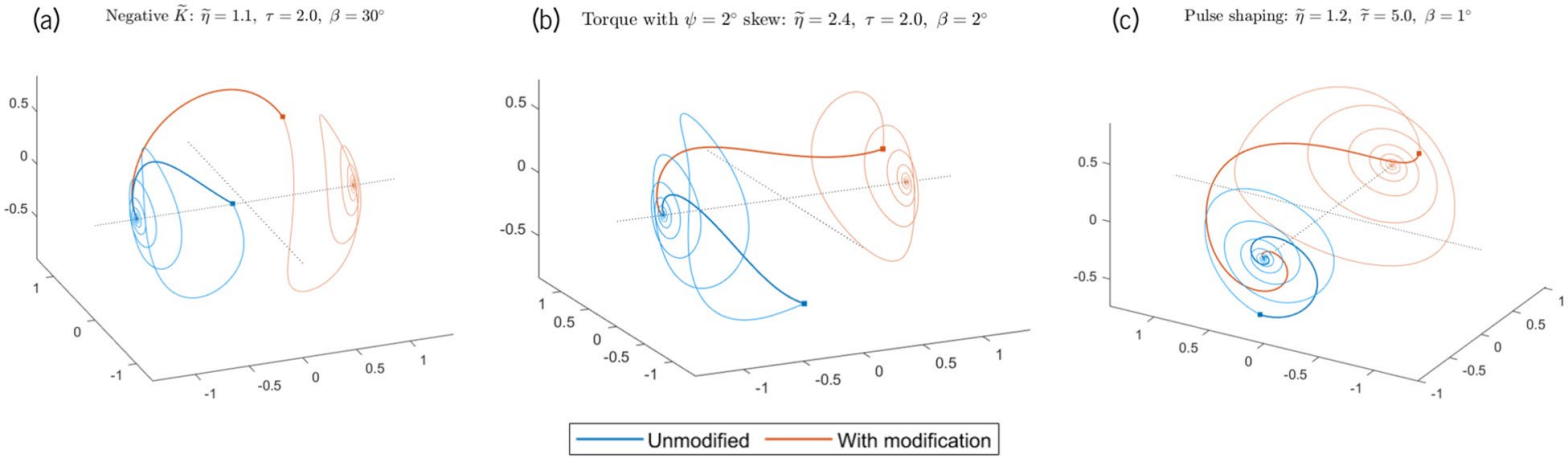
Unmodified and modified trajectories of the magnetization $\hat{m}$ for the three switching strategies discussed in the “Improving performance through zero-temperature effects” section: negative anisotropy, slightly out-of-plane torque, and pulse shaping. All trajectories are divided into two sections: $\hat{m}$ evolution with spin torque active (thicker lines), and $\hat{m}$ evolution after $\eta$ goes to zero (thinner lines). The changeover points, at $\widetilde{t} = \widetilde{\tau }$, are marked on the graphs. For all three cases, the effect employed allows $\hat{m}$ to switch to the opposite state, while the same parameter set ($\widetilde{\eta }$, $\widetilde{\tau }$, $\beta$) in an unmodified system leads to failed switching.

Pulses that end up in this chaotic region are most susceptible to manipulation of transient switching dynamics: the boundary between failed and successful switching is so marginal that small, otherwise-negligible effects can tip a particular pulse to one side or another. If we can tweak the spin pulse-driven magnetization trajectories, the speed of the magnetization along said trajectories, or the field-driven landscape during the no-current portion of the switching process, we can shrink, shift, or nullify this stochastic regime and thereby reduce the critical currents needed for safe and reliable SOT-MRAM switching. In the following sections, we present several alterations to fundamental materials, device architecture, and electrical inputs to help achieve this goal. The “Improving performance through zero-temperature effects” section covers modifications that function at zero temperature; the “Probabilistic switching through thermal effects” section introduces the concept of thermal noise fields and covers modifications to that new simulation landscape.

In the following sections, we split the switching process into two categories: the *steady-state energetics*, which are determined solely by the anisotropy of the magnetic layer and the total input energy from the spin current pulse, and the *transient dynamics*, which are determined by the effective fields and torques that only arise during the switching process and are not present in the steady-state. The latter portion includes demagnetization fields and field-like and anti-damping-like contributions from the spin transfer torque. Below, we consider strategies and mechanisms that only affect the transient dynamics, while keeping the steady-state energy landscape unchanged, and demonstrate that such interventions can produce significant improvements in switching efficiency.

Our analyses focus on *trajectories*, and forces and torques that “push” or “pull” the magnetization $\hat{m}$. We can also analyze this behavior from the standpoint of the energy landscape, and focus on transient changes to that landscape that “guide” $\hat{m}$ along the gradient from its start state to the end state. The latter is an entirely equivalent formulation, but we choose the former because it is more intuitively grasped and more amenable to our simulation framework.

## Improving performance through zero-temperature effects

**Figure 3:**
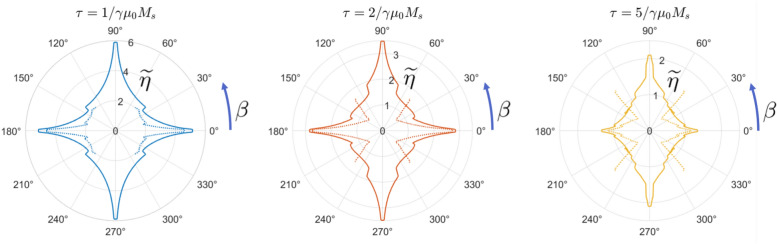
Nondimensionalized switching current $\widetilde{\eta }$, shown as the polar radius, for in-plane switching with an in-plane easy axis (solid lines) and an in-plane hard axis of equal magnitude (dotted lines). For certain pulse widths $\tau$ and polarization angles $\beta$, switching performance is improved significantly even though the in-plane energetic landscape remains unchanged. Note that in the negative-anisotropy case, $\beta$ values close to the hard axis lead to non-deterministic switching.

### Negative uniaxial magnetocrystalline anisotropy

We first tested the replacement of the unique in-plane easy axis with a unique in-plane *hard* axis. In mathematical terms, this is equivalent to adjusting the effective (nondimensionalized) anisotropy field $\widetilde{\textbf{B}}_{\text {ani}} = 2\frac{K}{\mu _0 {M_s}^2}(\hat{m} \cdot \hat{k})\hat{k}$ by making $\widetilde{K} \equiv K / \mu _0 {M_s}^2$ negative and placing $\hat{k}$ in the orthogonal in-plane direction.

If the magnitude of $\widetilde{K}$ is kept constant, the in-plane energy landscape is completely unchanged: the stable states for $\hat{m}$ remain the same, and so does the steady-state energetic barrier between the two states. Nevertheless, the sign of the anisotropy affects the transient dynamics of the system. During the transition between the stable states, the magnetization always picks up an out-of-plane component, and the demagnetization field arises to tamp it down, creating an additional barrier for the switching process. An in-plane easy axis produces effective anisotropy fields that work with the demagnetization to fight these $m_z$ components. An in-plane hard axis, on the other hand, works against and partially cancels out this demagnetization-related barrier. This additional “boost” during the switching process can be seen in Fig. 2a: precessing around the hard axis rather than the easy axis pushes $\hat{m}$ past an intermediary state and towards the final $+\hat{x}$ state more effectively within the same current pulse duration.

This phenomenon results in a clear and consistent decrease in the required switching current across a range of pulse widths and polarization angles. At polarization angles $\beta$ close to 45°—associated with large spin torques and high out-of-plane magnetization components—the switching current can be cut in half (Fig. 3). For larger $\beta$ closer to the hard axis, switching becomes non-deterministic: $\hat{m}$ is drawn too close to the in-plane hard axis and flung away, with its final position dependent entirely on the timing of the precessional process.

For $\beta$ less than around $50^{\circ }$ from the in-plane easy axis, the substitution of easy-axis anisotropy for orthogonal hard-axis anisotropy provides for significant switching benefits: less current over the same pulse width, or equivalently a shorter pulse with the same current magnitude. In both cases, we achieve successful switching with significantly less energy expended. To achieve these improvements, we require a magnetic material with negative uniaxial magnetocrystalline anisotropy. While this property is rare, it has been found (among other places) in the Co-Ir alloy system, with magnitudes up to $6 \times 10^{6}$ erg/cm^3^ at an Ir fraction of 0.20^18^. This is a magnitude comparable to well-known positive uniaxial anisotropy systems. Our experimental exploration of in-plane SOT-MRAM devices using this material system, which will enable us to validate these efficiency improvements in the lab, is ongoing.

### Slightly out-of-plane torque

**Figure 4:**
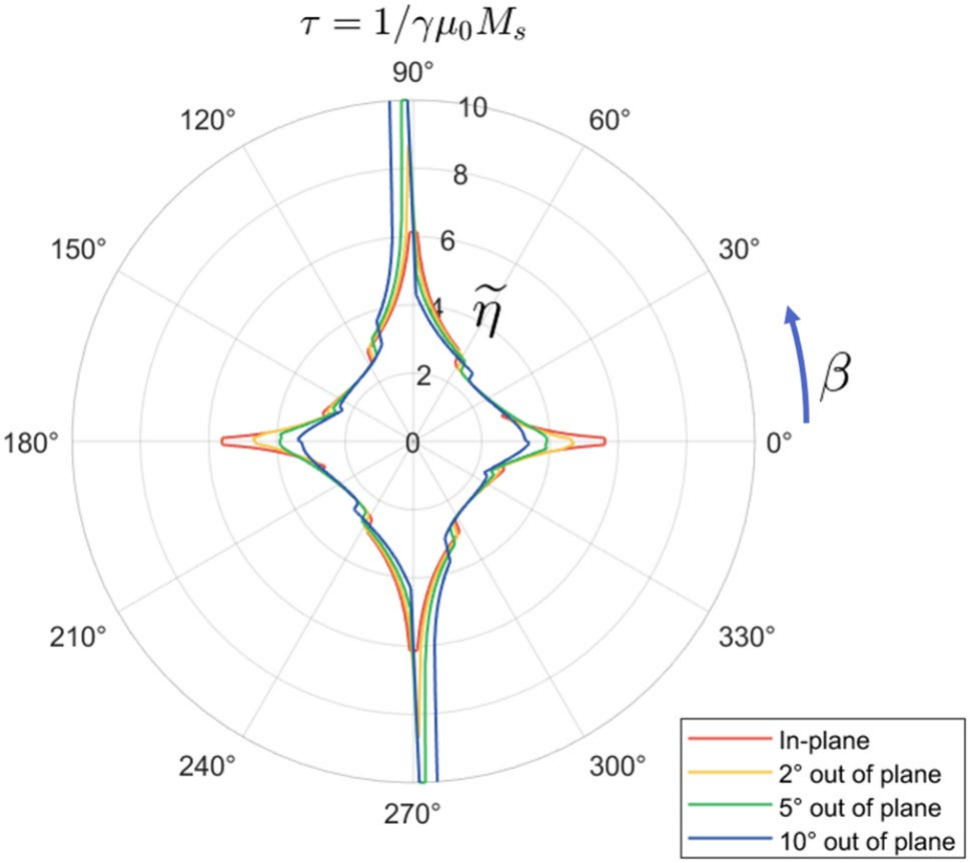
The critical current needed to switch from one in-plane magnetization state to the other, $\widetilde{\eta }$, as a function of the spin torque polarization angle $\beta$. Adding an out-of-plane skew angle to the spin torque greatly decreases the required switching current near $\beta = 0^{\circ },\ 180^{\circ }$ but makes switching impossible for a small region near the hard-axis.

We now turn to another modification of the SOT-MRAM switching process: a slight amount of out-of-plane torque injected in concert with the main in-plane torque component. Mathematically, this is equivalent to skewing the spin polarization vector out of the plane of the film, thereby changing it to

$$\hat{p} = \begin{bmatrix} \cos (\phi )\cos (\beta )\\ \cos (\phi )\sin (\beta )\\ \sin (\phi )\\ \end{bmatrix}$$

where $\beta$ is the in-plane polarization angle and $\phi$ is the out-of-plane skew angle. All other elements of the switching process, including the pulse shape, pulse duration, and anisotropy-derived energy landscapes, remain unchanged. The primary effect of this alteration is to modify the temporary and transient spin transfer torque terms found in the Landau-Lifshitz-Gilbert equation.

Like the in-plane axis of negative crystalline anisotropy covered above, this skewed torque helps counteract the transient effects of the demagnetization field during the switching process. The out-of-plane portion of $\hat{p}$ creates a “field-like” torque that partially cancels out the demagnetization arising from the out-of-plane component of $\hat{m}$. As shown in Fig. 4, the resulting reduction of the energy barrier decreases switching currents across a range of in-plane $\beta$.

The effect is further enhanced for $\beta$ close to the in-plane easy axis. Here, the main benefit derives from the addition of extra torque during the initial stages of the switching process. Depending on the particular values of $\phi$ and $\beta$, the skewing of $\hat{p}$ can more than double the $\hat{m} \times \hat{p} \times \hat{m}$-type torque at $t=0$, pushing the magnetization over towards the final state faster and further than in the $\phi = 0^{\circ }$ case. Figure 2b shows the effect of this additional torque—a slight $\psi = 2^{\circ }$ skew means that for the same input pulse, the trajectory $\hat{m}$ gets pushed much further along the *x*-axis with the same input current pulse, and this makes the difference between failed and successful switching. As can be seen in Fig. 4, however, this improvement is only prominent when $\beta$ is an order of $\phi$ or less away from the closest in-plane easy axis.

We furthermore note that for two limited regions of $\beta$ close to the in-plane hard axes, the addition of the polarization skew $\phi$ makes switching impossible. The reason for this surprising phenomenon lies in the interplay between the anisotropy and demagnetization fields. The equilibrium position for $\hat{m}$ during the application of the spin torque is $\hat{p}$; when the spin torque is turned off, the $m_x$-dependent anisotropy field usually takes over and moves $\hat{m}$ to the desired steady-state in-plane configuration. However, if $\hat{p}$ has a *z*-component, then the $m_z$-dependent demagnetization field activates as well. When the demagnetization term is greater than the anisotropy term—possible only for weak anisotropy constants and/or positioning of $\hat{p}$ such that $\cos \beta < \sin \phi$—then the final position of $\hat{m}$ is determined by its precession along $-m_z\hat{z}$. For the two slices of the polar plane seen in Fig. 4, the switching and precession chiralities are such that $\hat{m}$ returns to its original state instead of switching successfully. The set of pulses that satisfy all of these conditions is rather small, however, and the concomitant regime of failed switching can be avoided by a slight adjustment in skew angle, current direction, or anisotropy constant.

Methods of adding out-of-plane components to spin Hall torques have been studied extensively, primarily in the context of out-of-plane SOT-MRAM systems^19^. Proposed strategies include wedge-type device geometries^20^, interlayer exchange coupling^21^, chirality-dependent Dyaloshinski-Moriya-type interactions^22^, and the use of topological insulators^23^. Utilization of such techniques in the construction of in-plane SOT-MRAM devices can help attain the low-$\beta$ reductions in switching current discussed here.

### Pulse shaping

**Figure 5:**
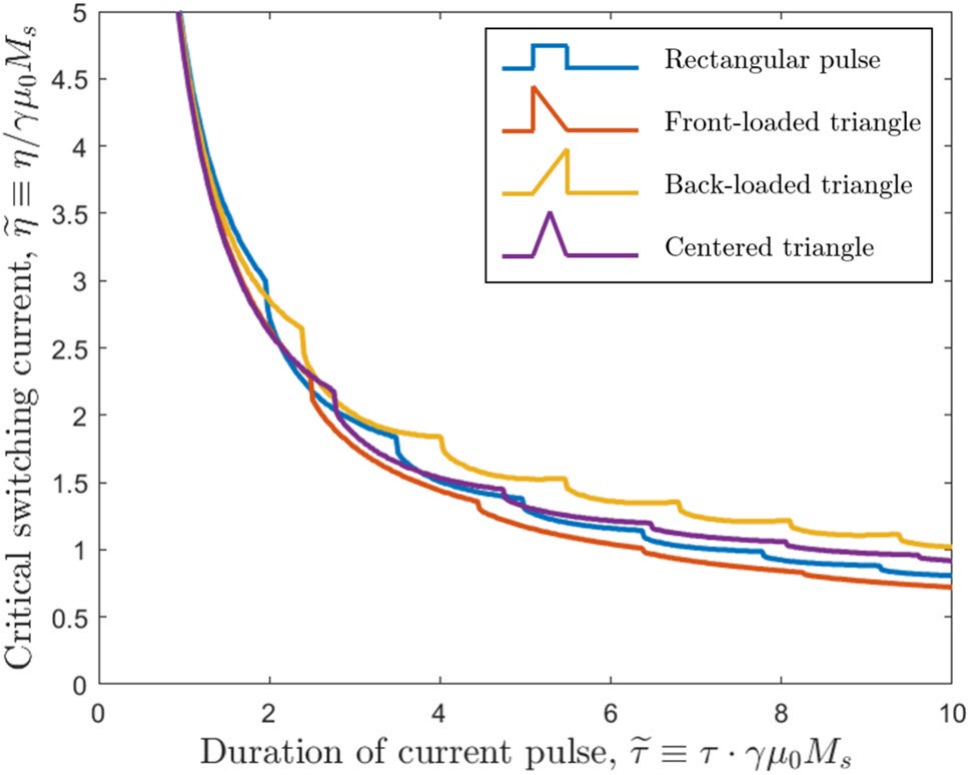
The nondimensionalized critical current needed to switch from one in-plane state to another, $\widetilde{\eta }$, as a function of pulse duration $\widetilde{\tau }$ for four different pulse shapes. All pulses have the same duration and the same total momentum transfer (i.e., the same area under the curve), but changing what proportion of the current is delivered at what time strongly affects the switching process. Of the four pulses shown here, the front-loaded pulse (red) leads to the lowest $\widetilde{\eta }$ for all $\widetilde{\tau }$, while the back-loaded pulse (yellow) leads to the highest.

We conclude this section by proposing a third method to improve the efficiency of spin Hall switching. This method does not require any changes to the materials or geometry of the MRAM device, as it relies solely on a change in the *input*, the current pulse. With this approach, problems associated with incorporating new materials or new device architectures into the material deposition and nanofabrication processes can be entirely avoided, and we can achieve improved efficiencies and writing speeds without making any changes to pre-existing device frameworks.

We keep the total injected momentum, $\int _0^{\infty } \widetilde{\eta }(\widetilde{t}) d\widetilde{t}$, constant. However, we change the shape of the pulse from the standard rectangle of magnitude $\widetilde{\eta }$ and duration $\widetilde{\tau }$ to triangular pulses with height $2\widetilde{\eta }$ and duration $\widetilde{\tau }$. We vary the position of the peak to create front-loaded, centered, and back-loaded triangles, as seen in the inset of Fig. 5.

All of these pulses have the same area under the curve, and therefore the same total energy added to the SOT-MRAM system by the spin pulse. However, their effects are quite different. In Fig. 5, we see that the front-loaded triangle provides the best switching efficiency, a clear improvement over the rectangular pulse and other triangle pulses over all pulse durations; the back-loaded triangle leads to the highest switching currents and worst efficiencies. We attribute this finding to the dynamics of the switching process. Initially, the magnetization needs a lot of torque to start precessing—enough to counteract the full force of the effective anisotropy torque, which is at its maximum at $t=0$. Once the magnetization is already moving, however, the effective torques pulling it back decrease, and keeping the spin torque constant at its $t=0$ value causes the majority of the input torque to be wasted. Therefore, the most efficient modulation of the incoming torque is one that starts off high and decreases throughout the switching cycle. Among the four pulse shapes shown in Fig. 5, the front-loaded triangle comes closest to this ideal, leading to the best performance. An example of this performance improvement is shown in Fig. 2c, in which we replace a rectangular pulse with a front-loaded triangle. Here, we observe that the triangle’s higher torques at the start of the switching process cause greater rotation and movement in $\hat{m}$, while the concomitant smaller torques at the end of the pulse cycle—after the magnetization has already gained momentum and crossed the *x*-axis—do not negatively affect switching in any way.

We note that an exhaustive simulation of all possible triangle pulses reveals that placing the triangle peak between $\widetilde{t} = 0.05\widetilde{\tau }$ and $\widetilde{t} = 0.2\widetilde{\tau }$ optimizes the switching current; the exact optimal peak position depends on the value of $\widetilde{\tau }$. Fourier-transform or variational-calculus approaches might enable us to find the global-optimum pulse shape for a given system configuration or set of inputs, but such investigation is beyond the scope of this study. With triangle pulses alone, moreover, we can achieve a significant and sustained improvement in SOT-MRAM switching current with no architectural or material adjustments to the device.

It is important to note that we have only considered the effect of pulse shaping on the spin torque terms of the Landau-Lifshitz-Gilbert equation. Unlike the other two mechanisms explored in this section, this strategy also has a secondary effect: changing the input current alters the time-evolution of the device temperature, which in turn affects the switching dynamics of the magnetization trajectory (as seen in the “Probabilistic switching through thermal effects” section). We have separated the two effects by forcing a temperature of $T = 0$ for all pulse widths, currents, and shapes. Allowing the time evolution of temperature to be affected by the current pulse will affect the results of this section, but since it is a higher-order phenomenon, the modifications will be minor in most cases. A full model of the connection between current and device temperature is beyond the scope of this paper, but it is a promising direction for future simulation research on this SOT-MRAM system.

## Probabilistic switching through thermal effects

**Figure 6:**
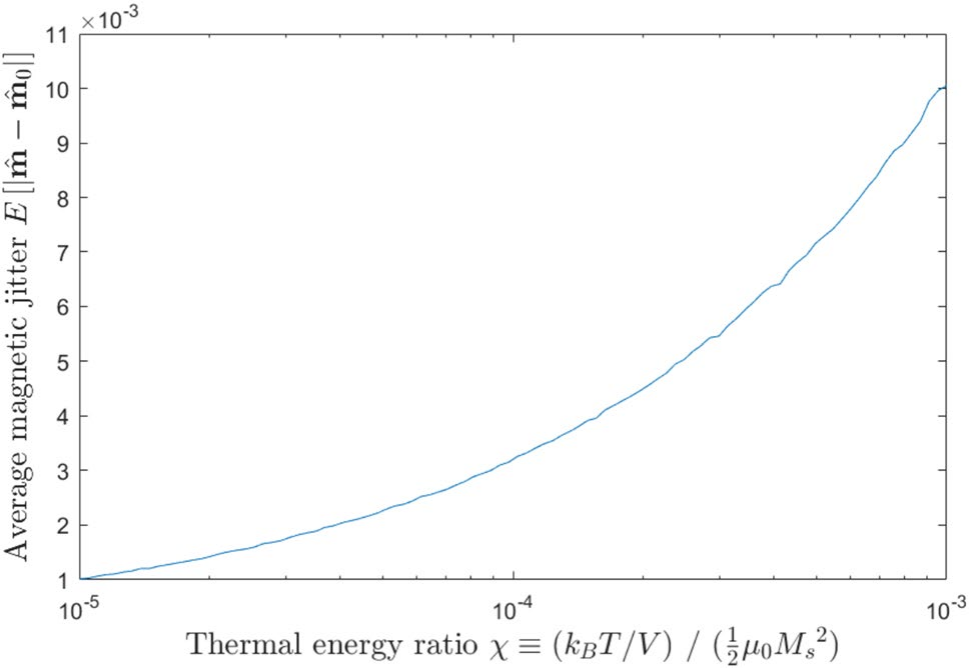
The monotonic exponential increase in magnetic jitter, found as the average deviation of $\hat{m}$ from its starting point, as we increase the ratio between thermal energy and magnetic self-energy, $\chi$.

Thus far, we have considered deterministic versions of the Landau-Lifshitz-Gilbert equation, in which the position of the magnetic moment is affected only by the fields and torques incumbent upon it. In such frameworks, if $\hat{m}$ damps to its lowest-energy configuration, it will stay there forever. When we add in thermal noise, this is no longer the case: $\hat{m}$ is perpetually perturbed in random directions by effective thermal fields, both during the switching process and afterwards. We call this phenomenon “magnetic jitter”. What is the effect of these random perturbations on the switching and stability of the in-plane SOT-MRAM magnetization?

### Modeling of thermal behavior

In order to include the magnetic jitter caused by thermal fluctuations, we follow the work of Brown^24^ and employ a Gaussian white noise “thermal field” $\vec {b}$ that increases in variance with the temperature *T*:

2 $$\begin{aligned} \langle b(t_1)b(t_2) \rangle = \dfrac{2k_B T\alpha }{V\gamma M_s}\delta (t_2 - t_1) \end{aligned}$$

The presence of the Dirac delta renders this field impossible to simulate numerically, so we discretize it using timesteps $\Delta t$. At the *i*th interval, we calculate an *average* thermal field $B_T^{(i)} \equiv \frac{1}{\Delta t} \int _{i \cdot \Delta t}^{(i+1)\cdot \Delta t} \vec {b}(t) dt$. The autocorrelation function of this new field is

$$\begin{aligned} \langle B_T^{(i)} B_T^{(j)}\rangle = \dfrac{1}{(\Delta t)^2} \int _{i \cdot \Delta t}^{(i+1)\cdot \Delta t} \int _{j \cdot \Delta t}^{(j+1)\cdot \Delta t} \langle b(t') b(t'')\rangle dt'' dt \end{aligned}$$

Plugging in (2) yields

3 $$\begin{aligned} \langle B_T^{(i)}B_T^{(j)} \rangle = \dfrac{2k_B T \alpha }{\gamma M_s V(\Delta t)^2} \int _{i\cdot \Delta t}^{(i+1)\cdot \Delta t} \delta _{ij} dt' = \dfrac{2k_B T \alpha }{\gamma M_s V \Delta t}\delta _{ij} \end{aligned}$$

Equation (3) represents a discrete Gaussian random variable with zero mean and variance $\sigma ^2 = 2k_B T \alpha / \gamma M_s V\Delta t$, describing the random thermal field $\vec {B_T}$ seen by the magnetic moment $\hat{m}$. At each timestep $\Delta t$, $\vec {B_T}$ goes to a different uniformly random direction and takes on a magnitude selected from the normal distribution shown above.

To nondimensionalize, we define a nondimensional timestep, $\delta \equiv \Delta t \cdot \gamma \mu _0 M_s$, yielding

$$\begin{aligned} {\sigma _B}^2 = \dfrac{2 k_B T \alpha }{V}\dfrac{\mu _0}{\delta } = \dfrac{\alpha }{\delta } \dfrac{k_B T / V}{\frac{1}{2}\mu _0 {M_s}^2}(\mu _0M_s)^2 \end{aligned}$$

We now define $\chi \equiv (k_B T / V) / (\frac{1}{2}\mu _0 {M_s}^2)$. We will call it the *thermal parameter*, a *ratio* between the thermal energy density and magnetic (self-)energy density in the cell. For the smallest magnetic cells at elevated temperatures, it can rise to the order 10^-2^; for large cells at room temperature, it can drop to 10^-5^. With this substitution, the standard deviation of the nondimensionalized field $\widetilde{\textbf{B}}_T$ becomes

4 $$\begin{aligned} \widetilde{\sigma _B} = \frac{\sigma _B}{\mu _0 M_s} = \sqrt{\dfrac{\alpha \chi }{\delta }} \end{aligned}$$

This is the variance of the random nondimensionalized field $\widetilde{\textbf{B}}_T$ that is added into the $\widetilde{\textbf{B}}$ of (1). At each timestep $\delta$, we calculate a new $\widetilde{\textbf{B}}_T$ with the corresponding Gaussian-random magnitude and a uniform random direction. This in turn causes a $\hat{m} \times \vec {B}$-type torque expressed in the LLG equation.

Since the direction of the thermal field changes with each timestep, there is an infinitesimally low probability that these terms can single-handedly cause a large movement like a wholesale switch in $\hat{m}$. However, as seen below, the small perturbations arising from thermal fields do play a significant role in the switching process, especially in chaotic regimes highly susceptible to initial conditions.

### Magnetic jitter and stability

To measure the effect of thermal fields and quantify the effect of the thermal ratio parameter $\chi$, we begin by considering a simple system: a magnetization in its equilibrium in-plane state, unaffected by spin torque and held in place by uniaxial anisotropy. At $t=0$, we place $\hat{m}$ exactly along the anisotropy axis, and then average its subsequent displacement from its starting position—i.e., $E\left[ |\hat{m}(t) - \hat{m}(t = 0)| \right]$ over a long time window. The resulting standard deviation is our metric for magnetic jitter. Just as in the $T=0$ case, the *average* position of $\hat{m}$ is still the equilibrium in-plane position—thermal fields do not induce a particular bias towards any given direction. However, $\hat{m}$ is, at any given time, displaced from that position by a vector whose absolute magnitude varies with temperature, and this absolute displacement is what we call “jitter”. Higher temperatures cause larger thermal fields and consequently larger shifts in magnetization. As shown in Fig. 6, we find that rising temperature monotonically increases the jitter in $\hat{m}$, but the effect is relatively limited over two orders of magnitude in $\chi$.

### Probabilistic switching with alleviation of stochasticity

**Figure 7:**
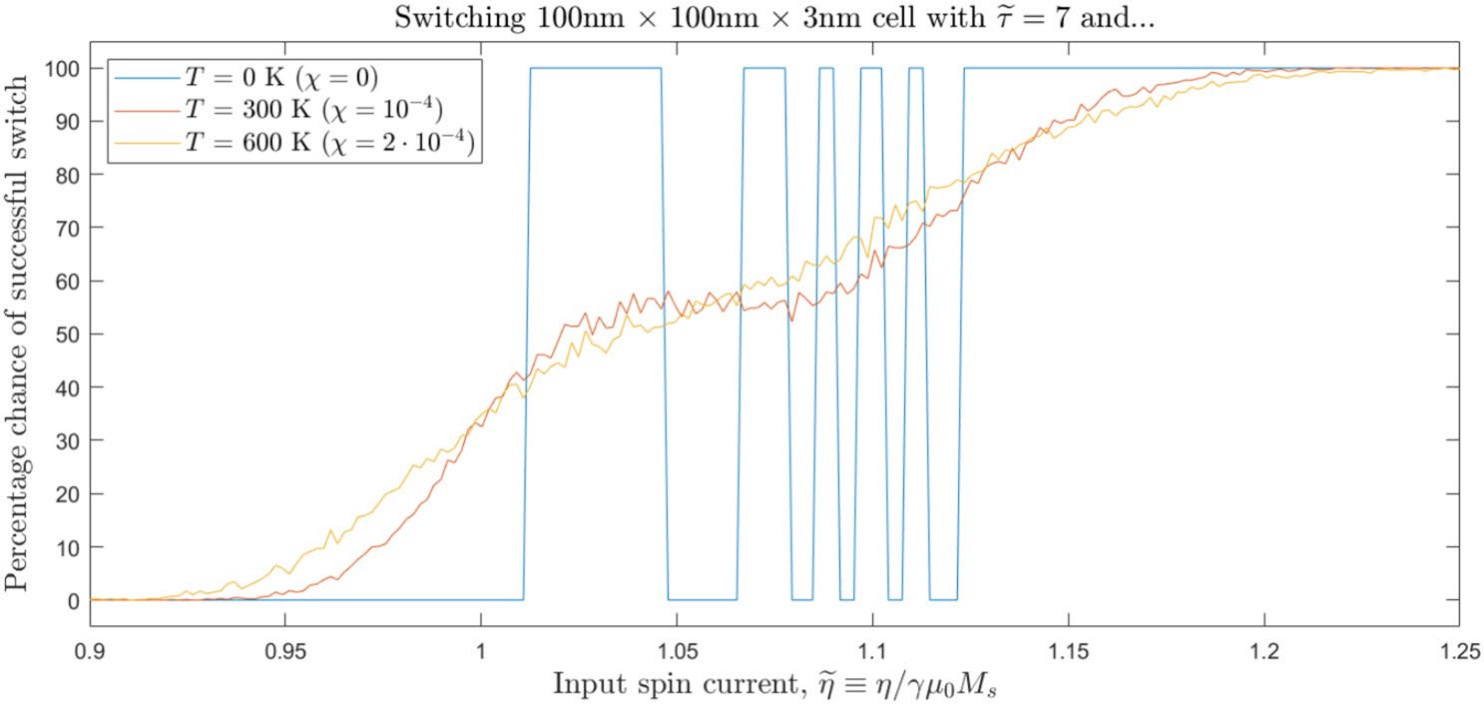
The probability of SOT-MRAM switching as a function of nondimensional spin currents $\widetilde{\eta }$ and pulse durations $\widetilde{\tau }$, at different temperatures *T*. Elevated temperature “smears out” the former stochastic regime, but beyond a certain point *T* has little effect.

We now move on from the effect of thermal fluctuations on stationary magnetizations to investigate the effect of thermal fields on magnetizations during the spin-torque-driven SOT-MRAM switching process. In previous simulations, the trajectory of $\hat{m}$ given certain pulse conditions and effective fields could be found deterministically, even in the so-called “chaotic regions” where these trajectories were highly susceptible to small changes in $\eta$, $\tau$, or $\beta$. Now, however, thermal fluctuations introduce a fundamental layer of randomness to the problem, and two different simulations with the same inputs but different random thermal fields can produce completely different magnetization trajectories. Therefore, we must shift our fundamental metric from a binary classification between failed and successful switching to a *probability* of switching derived from an ensemble of simulation results.

**Figure 8:**
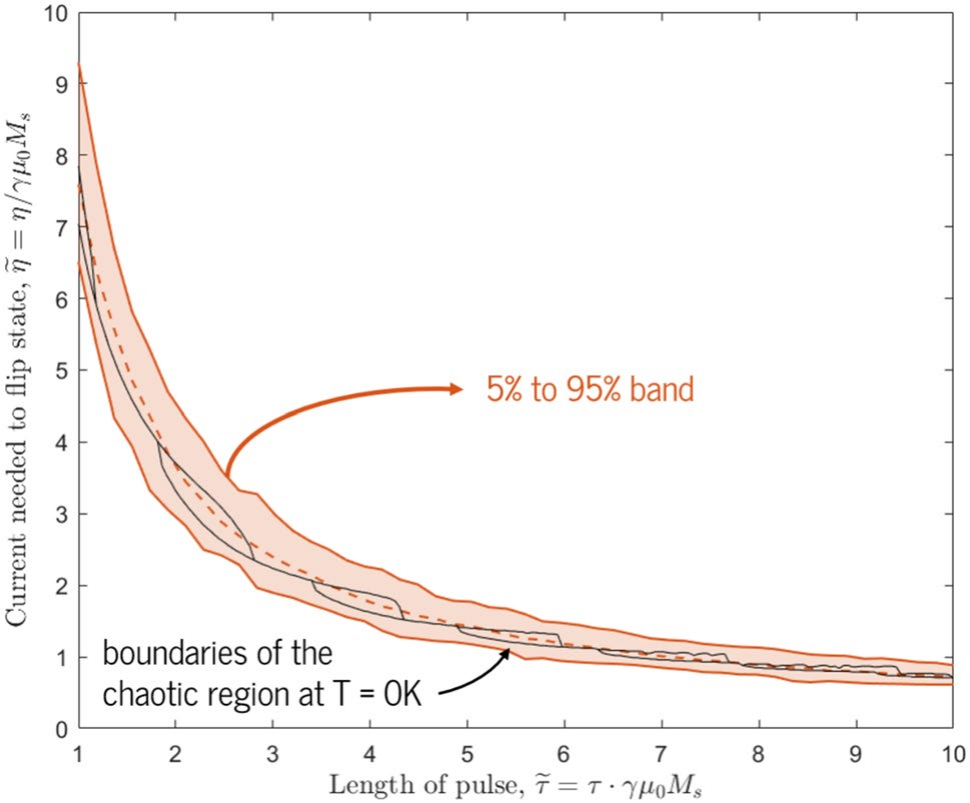
The critical switching currents $\widetilde{\eta }$ needed for pulse widths $\widetilde{\tau }$ at thermal parameters $\chi = 0$ and $\chi = 10^{-3}$. The $\chi = 0$ switching boundaries are shown in black; the chaotic regime exists between the two boundary lines. At elevated temperature, we replace hard boundaries with a smooth transition band between failed and successful switching, shown in red; the bottom solid line represents a 5% chance of switching, the middle dotted line represents a 50% chance, and the top solid line represents a 95% chance. The width of this transition band depends on temperature (i.e., on $\chi$), but its center point remains stable.

When we add thermal fluctuations to the switching model, we find that abrupt changes between failed and successful switching regimes are replaced by smooth transitions in the probability landscape. As seen in Fig. 7, the $\chi$-dependent term “smears out” the $T=0$ precession-dependent transitions between failed switching, successful switching, failed switching again, and a final band of successful switching; these are instead replaced by a monotonic increase in the probability function from 0% to 100%.

The extent of the transition region and the smoothness of the resulting curve are deeply affected by the value of $\chi$. However, for a given device volume and saturation magnetization, $\chi$ varies linearly in cell temperature, and the thermal field that it causes, $\widetilde{\textbf{B}}_T$, goes only as the square root of *T*. This relationship blunts the effect of even large changes in environmental temperature. In Fig. 7, for instance, doubling the device temperature from 300 K to 600 K only slightly affects the probabilities of SOT-MRAM switching. We can also change $\chi$ by altering the volume of the magnetic cell: since $\chi \sim 1/V$, decreasing the cell radius at constant temperature leads to a quadratic increase in the thermal parameter.

We now return to our detailed studies of switching behavior for a range of different current pulses and durations, using rectangular current pulses throughout. An exhaustive simulation of the threshold switching currents that the previous three-way distinction between failed, successful, and stochastic intermediate regimes is replaced with a smooth “transition band” at elevated temperatures. In Fig. 8, we plot lines representing a 5%, 50%, and 95% chance of switching, and note that these lines never cross (i.e., the currents associated with these lines always maintain the same order with respect to each other for all pulse widths). For all $\widetilde{\tau }$ values, therefore, we see the same sort of monotonic probability increase seen in Fig. 7. We also find that the 50% line is in the middle of the zero-temperature stochastic region, and that the entire $T=0$ stochastic region is encapsulated in the room-temperature transition band. We note that as we elevate the thermal parameter $\chi$, the width of the transition band increases, but its central (50%) line stays in the same place.

The reason for this new, probabilistic switching behavior is simple. Under the influence of randomized thermal fields, the single predetermined magnetization trajectory assumed in zero-temperature models bifurcates into an infinite number of possible paths. Even though the thermal field is relatively small in magnitude, the resulting deviations in trajectory are massive: a minor perturbation at the very beginning of the switching cycle, when all other fields and torques are minimal, can cause completely different behavior down the line. When the current pulse cuts off, the nondeterministic nature of the trajectory means that we cannot know where $\hat{m}$ ends up—and whether it is in a region where it will damp to the final state or be flung back to its initial position. Of course, all potential spin pulse-driven trajectories must start and end in the same place. Therefore, if we consider a sufficiently short or sufficiently long pulse width, we can be almost entirely assured that the switching process will fail or succeed (respectively). In between these two extremes, however, the probabilistic nature of the process holds sway. This basic understanding also applies when we elevate the ambient temperature for all the modified zero-*T* scenarios covered in the “Improving performance through zero-temperature effects” section above; the exact functional dependences of the resulting changes are beyond the scope of this study.

In recent years, a growing body of literature has explored the use of magnetic randomness to create so-called “probabilistic bits” (p-bits)^25^. These bits, used in the probabilistic computing framework, have the potential to simulate quantum processes and run efficient and fast algorithms for integer factorization and other classically-hard computing problems^26^. In effect, they can realize at least some of the promise of quantum computing without the need for specialized, low-temperature hardware. Current work in this field focuses on either asynchronous thermal-field-driven fluctuations^27^ in magnetic free layers or on STT-MRAM switching with precisely-chosen input currents^28^. Our work suggests an alternative p-bit methodology based on the SOT-MRAM framework. Using the devices we propose here, we can create a p-bit whose probability can be tuned at will between 0% and 100% through the electrical pulse inputs $\widetilde{\eta }$ and $\widetilde{\tau }$, without deleterious sensitivity to external parameters such as the device temperature. We can generate these probabilities quickly, at sub-nanosecond timescales, and unlike in previous work, we do not have to compromise on the stability of the eventual written state to achieve this. Finally, we can trigger the creation of a new random bit at will through the introduction of a pulse, an improvement over unstable MTJs that switch at unpredictable times^27^ or STT-MRAM bits that require resets due to the P $\longrightarrow$ AP asymmetry^28^. These three attributes make the SOT-MRAM p-bit presented here attractive for future study and development in the realm of probabilistic computing.

### Anisotropy curve shaping

**Figure 9:**
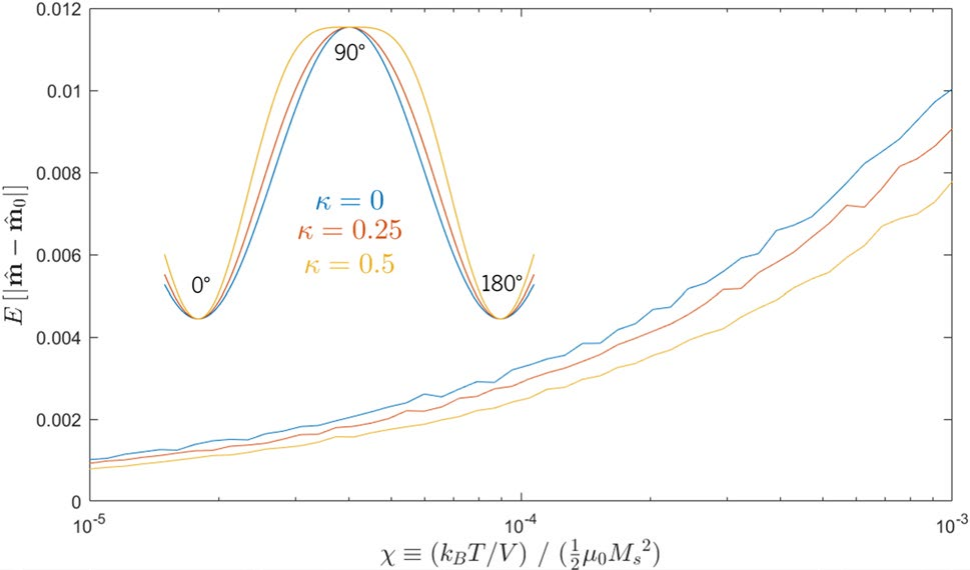
Inset: “shaping” the anisotropy curve by varying $\kappa$, the ratio between the first- and second-order terms. A higher $\kappa$ means a steeper climb between easy-axis and hard-axis states, even as the barrier energy stays constant. Main figure: the effect of $\kappa$ on the magnetic jitter. A higher $\kappa$ creates higher effective anisotropy fields, thereby suppressing thermal fluctuation in the magnetization.

We conclude this paper by demonstrating a final strategy to alter the switching landscape. Unlike the other strategies previously described in the “Improving performance through zero-temperature effects” section, this strategy does not aim to improve a switching property, like the critical current $\widetilde{\eta }_{\text {crit}}$, but rather a *thermal* property: the magnetic jitter.

Here, we adjust the anisotropy energy function to include a negative second-order (sin^4^) term:

$$\begin{aligned} \mathscr {E}_{\text {ani}}(\varphi ) = K_1\frac{\sin ^2\varphi - \kappa \sin ^4\varphi }{1-\kappa } \end{aligned}$$

where $\varphi$ is the angle between the in-plane magnetization and the in-plane easy axis and $\kappa$ is defined as the ratio between the first-order and second-order anisotropy terms. The associated effective field, generalizing to an arbitrary anisotropy axis $\hat{k}$ and an arbitrary magnetization $\hat{m}$ in 3D space, is

$$\begin{aligned} \widetilde{\textbf{B}}_{\text {ani}} = \frac{2K_1}{\mu _0{M_s}^2}\left( \left( \frac{1}{1-\kappa } - \frac{2\kappa }{1-\kappa }\right) (\hat{m} \cdot \hat{k}) + \frac{2\kappa }{1-\kappa }(\hat{m}\cdot \hat{k})^2\right) \end{aligned}$$

The energy barrier between the hard-axis and easy-axis states is always $K_1$, irrespective of $\kappa$, but the shape of the curve changes to create a steeper climb to the hard-axis state, as shown in the inset of Fig. 9. Altering the shape of the anisotropy landscape in this way creates a higher effective field preventing $\hat{m}$ from leaving an equilibrium state, while preserving the energy barrier required to switch from one equilibrium state to the other. This in turn creates less magnetic jitter for the same switching current in a SOT-MRAM device. There is, however, a natural limit on $\kappa$—beyond $\kappa = 0.5$, metastable high-energy states develop at 90°and 270°, complicating the operation of the SOT device.

This effect can be seen in Fig. 9, in which the magnetic jitter (as measured by the average absolute displacement of the magnetization from the lowest-energy state) decreases monotonically with increasing $\kappa$. At the same time, the switching characteristic displayed in Fig. 8 does not change at all—the current needed to ensure 50% probability of switching stays in the same place irrespective of $\kappa$, with only small variations in the 5% and 95% lines due to statistical noise.

These simulation results demonstrate the potential for magnetic memory devices to gain additional thermal stability without affecting critical switching currents. We note that the anisotropy characteristics needed to enable this behavior cannot be derived through device geometry: it is known that the demagnetization-type fringing fields that lead to shape anisotropy can only generate an isotropic or uniaxially-anisotropic landscape in the plane of the film. Therefore, the second-order anisotropy term must be crystallographic in nature. Non-negligible second-order anisotropy contributions are present in many common ferromagnetic materials, including iron and cobalt^29^, but they usually do not oppose the sign of the first-order contribution $K_1$. Some materials, however, do exhibit opposite signs for $K_1$ and $K_2$, including Co-alloyed magnetite^30^ and low-temperature europium sulfide^31^. Unfortunately, the energetic landscapes in these materials are not uniaxial in nature, and they also do not exhibit positive first-order anisotropy. Further experimental research is required to engineer the anisotropy structures that can enable the improvements covered in this section.

## Conclusion

The transient dynamics of the SOT-MRAM switching process are complex. Understanding them requires a coherent theory for two interrelated phenomena: the direct driving of magnetization trajectories by input spin pulses and the spin-torque-free precession of those magnetizations under the influence of effective anisotropy and demagnetization fields. Both of these factors lead to intricate, whirling regime boundaries, and their combination leads to a quasi-stochastic region of switching. It is this stochastic regime that offers the most dynamic and useful levers of control for SOT-MRAM switching. In this work, we have shown that slight adjustments in the injected spin torque, effective field landscape, or timing of the transient forces—achieved through simple tweaks to device geometry, material architecture, or electrical inputs—can shrink or shift the stochastic regime, thereby enabling substantial improvements in writing speed or efficiency within the well-known SOT-MRAM framework. Furthermore, we have demonstrated that at high temperature, the stochastic regime collapses into a set of well-defined and tunable bands of pure probabilistic switching, allowing for the creation of probabilistic bits triggered and operated by spin torque pulses. These new p-bits, which are orders of magnitude faster and more tunable than the current state of the art, represent a powerful new pathway for probabilistic computing with magnetic devices. These exciting results are enabled by our theoretical grounding in the transient mechanics of this magnetic system, and further study of its fundamental theory can enable even greater successes in the future.

## Supplementary Information

Supplementary Information 1.

Supplementary Information 2.

## Data Availability

All datasets generated and analyzed in this work are included in the supplementary information files of this manuscript.
