# Supplementary material for: Manipulating transient SOT-MRAM switching dynamics for efficiency improvement and probabilistic switching
Source: Sci Rep. 2025 Oct 31;15:38182. doi: 10.1038/s41598-025-22014-1 (PMC12578803; doi:10.1038/s41598-025-22014-1)
Supplement: Supplementary file 2 — Supplementary Information 2. [file 41598_2025_22014_MOESM2_ESM.pdf]

Critical spin-current parameters for in-plane SOT-MRAM switching: theoretical derivation

Shreyes Nallan (shreyes@cmu.edu) and Jian-Gang Zhu, Carnegie Mellon University

The nondimensionalized Landau-Lifshitz-Gilbert equation with Slonczewski's modification for spin transfer torque reads

$$\frac{d\hat{m}}{d\widetilde{t}} = -\frac{1}{1+\alpha^2}\left(\hat{m}\times\left[\widetilde{\mathbf{B}} + \alpha\hat{m}\times\widetilde{\mathbf{B}} - \alpha\widetilde{\eta}\hat{p} - \widetilde{\eta}\hat{p}\times\hat{m}\right]\right)$$

where $\widetilde{\mathbf{B}} \equiv \vec{B}/\mu_0 M_s$, $\widetilde{\eta} \equiv \eta/\gamma\mu_0 M_s$, and $\widetilde{t} \equiv t\cdot\gamma\mu_0 M_s$.

We consider a spin injection in the form of a rectangular pulse: $\widetilde{\eta}(\widetilde{t}) = \widetilde{\eta}$ for $\widetilde{t} < \widetilde{\tau}$ and $\widetilde{\eta}(\widetilde{t}) = 0$ afterwards. We can therefore split the magnetization trajectory into two timespans: η-driven and η-free.

We will ignore the demagnetization effective field (or, alternately, posit large anisotropy constant $\widetilde{K}$ such that it overpowers the demagnetization $\forall t$). We will also choose the axis of in-plane anisotropy to lie along the x-axis. Then the nondimensionalized field is in one direction only: $\widetilde{\mathbf{B}} = 2\widetilde{K}m_x\hat{x}$, where $\widetilde{K} \equiv K/\mu_0 {M_s}^2$.

1 η-free trajectories

If $\eta = 0$, we have

$$\frac{d\hat{m}}{d\widetilde{t}} = -\frac{1}{1+\alpha^2}\left(\hat{m}\times\left[\widetilde{\mathbf{B}} + \alpha\hat{m}\times\widetilde{\mathbf{B}}\right]\right)$$

With our effective anisotropy field,

$$\hat{m}\times\widetilde{\mathbf{B}} = 2\widetilde{K}(m_x m_z\hat{y} - m_x m_y\hat{z})$$

$$\hat{m}\times\hat{m}\times\widetilde{\mathbf{B}} = 2\widetilde{K}\left[-m_x({m_y}^2 + {m_z}^2)\hat{x} + {m_x}^2 m_y\hat{y} + {m_x}^2 m_z\hat{z}\right]$$

If we select the x-component of this differential equation alone:

$$\frac{dm_x}{d\widetilde{t}} = -\frac{1}{1+\alpha^2}\left(\alpha\cdot -m_x({m_y}^2+{m_z}^2)\right)$$

Since $\hat{m}$ is by definition a unit vector, ${m_x}^2 + {m_y}^2 + {m_z}^2 = 1$, and

$$\frac{dm_x}{d\widetilde{t}} = \frac{\alpha}{1+\alpha^2}m_x\left(1 - {m_x}^2\right)$$

This is a separable ODE in terms of $m_x(\widetilde{t})$ alone. Its solution is

$$m_x(\widetilde{t}) = \pm\frac{e^{\frac{\alpha}{1+\alpha^2}\widetilde{t}}}{\sqrt{e^{\frac{2\alpha}{1+\alpha^2}\widetilde{t}} + \left(\frac{1}{{m_{x_0}}^2} - 1\right)}}$$

where m_{x_0} is the initial condition of m_x. This is a sigmoid-type function that starts at m_{x_0} and ends at ± 1. The final state is entirely determined by the sign of m_{x_0} – i.e., by where the magnetization is left off at the time the current pulse ends. Please note that as can be seen in the main manuscript, this is *not* the case when we include the effects of demagnetization fields or thermal fluctuations: this is information that we have lost in this simplified system model.

2 η-driven trajectories

In this model, the final state of $\hat{m}$ is determined entirely by whether $m_x > 0$ at the time the $\eta = 0$ portion of the trajectory begins. Therefore, if we now find the trajectory of $\hat{m}$ when it is driven by spin current $\widetilde{\eta}$, and find the value of m_x when $\widetilde{t} = \widetilde{\tau}$, we will know whether a certain $(\widetilde{\eta}, \widetilde{\tau})$ current pulse leads to successful magnetization switching.

We now make several further assumptions. First, that $\alpha \ll 1$, or at least that it is significantly smaller than $\widetilde{\eta}$, to the extent that we can ignore the α-denominated damping terms. (This is not ideal because we want to damp the magnetization to a stable state, but we do allow damping to occur in the second η-free time segment, so the results should be qualitatively accurate, even if the actual quantitative results are off.)

Second, we assume an entirely in-plane spin polarization: $\hat{p} = \langle \cos\beta, \sin\beta, 0\rangle$. The LLG equation then simplifies to

$$\frac{d\hat{m}}{d\tilde{t}} = -\frac{1}{1+\alpha^2}\left(\hat{m}\times\widetilde{\mathbf{B}} - \widetilde{\eta}\,\hat{m}\times\hat{p}\times\hat{m}\right)$$

The first term has been calculated above. The second is

$$\hat{m}\times\hat{p}\times\hat{m} = \hat{m}\times\left[m_z\sin\beta\,\hat{x} - m_z\cos\beta\,\hat{y} + (m_y\cos\beta - m_x\sin\beta)\hat{z}\right]$$

$$= \left[{m_y}^2\cos\beta - m_x m_y\sin\beta + {m_z}^2\cos\beta\right]\hat{x} + \left[{m_z}^2\sin\beta - m_x m_y\cos\beta + {m_x}^2\sin\beta\right]\hat{y} + \left[-m_x m_z\cos\beta - m_y m_z\sin\beta\right]\hat{z}$$

And so we end up with a system of three coupled non-linear ODEs:

$$\frac{dm_x}{d\tilde{t}} = \frac{\widetilde{\eta}}{1+\alpha^2}\left[\left({m_y}^2 + {m_z}^2\right)\cos\beta - m_x m_y\sin\beta\right]$$

$$\frac{dm_y}{d\tilde{t}} = -\frac{1}{1+\alpha^2}2\widetilde{K}m_x m_z + \frac{\widetilde{\eta}}{1+\alpha^2}\left[\left({m_x}^2 + {m_z}^2\right)\sin\beta - m_x m_y\cos\beta\right]$$

$$\frac{dm_z}{d\tilde{t}} = \frac{1}{1+\alpha^2}2\widetilde{K}m_x m_y - \frac{\widetilde{\eta}}{1+\alpha^2}\left[m_x m_z\cos\beta + m_y m_z\sin\beta\right]$$

There is no simple, closed-form solution to this system of differential equations. What we will do instead is "bootstrap" an answer that approximates the true solution for small t. Given the initial condition $\hat{m}(t = 0) = -\hat{x}$, we make a first pass on the y ODE, setting the other m components to their initial values $m_x = -1$ and $m_z = 0$:

$$\frac{dm_y}{d\tilde{t}} = \frac{\widetilde{\eta}}{1+\alpha^2}\left[\sin\beta + m_y\cos\beta\right]$$

This is a simple first-order ODE, and its solution is an exponential:

$$m_y(\tilde{t}) = \tan\beta\,(e^{\frac{\widetilde{\eta}}{1+\alpha^2}\cos\beta\,\tilde{t}} - 1)$$

This approximation does not work for β near 90°, because the tangent goes to infinity. It also does not work for large t, because the exponential increases without bound and eventually produces a nonphysical $|m_y| > 1$. This result only holds for small t (i.e., at the very beginning of the switching process).

We now plug this m_y back into the ODE for m_x. We will keep the approximations ${m_y}^2 = {m_z}^2 = 0$ in the first term, but substitute the m_y in the second term, as follows:

$$\frac{dm_x}{d\tilde{t}} = -\frac{\widetilde{\eta}\sin\beta}{1+\alpha^2}\tan\beta\,(e^{\frac{\widetilde{\eta}}{1+\alpha^2}\cos\beta\,\tilde{t}} - 1)m_x$$

This, too, is a separable ODE:

$$\int\frac{1}{m_x}\,dm_x = -\frac{\widetilde{\eta}}{1+\alpha^2}\frac{\sin^2\beta}{\cos\beta}\int(e^{\frac{\widetilde{\eta}}{1+\alpha^2}\cos\beta\,\tilde{t}} - 1)\,d\tilde{t}$$

$$\ln m_x = \frac{\widetilde{\eta}}{1+\alpha^2}\frac{\sin^2\beta}{\cos\beta}\left[\tilde{t} - \frac{1+\alpha^2}{\widetilde{\eta}\cos\beta}\,e^{\frac{\widetilde{\eta}}{1+\alpha^2}\cos\beta\,\tilde{t}}\right] + C$$

$$m_x = C\exp\left(\frac{\widetilde{\eta}}{1+\alpha^2}\frac{\sin^2\beta}{\cos\beta}\left[\tilde{t} - \frac{1+\alpha^2}{\widetilde{\eta}\cos\beta}\,\exp\left(\frac{\widetilde{\eta}}{1+\alpha^2}\cos\beta\,\tilde{t}\right)\right]\right)$$

$$= C\exp\left(\frac{\widetilde{\eta}}{1+\alpha^2}\sin\beta\tan\beta\,\tilde{t}\right)\exp\left(-\tan^2\beta\exp\left[\frac{\widetilde{\eta}\cos\beta}{1+\alpha^2}\,\tilde{t}\right]\right)$$

Solving for the initial condition, $m_x(0) = -1$, yields

$$m_x(\tilde{t}) = -\exp\left(\frac{\widetilde{\eta}}{1+\alpha^2}\sin\beta\tan\beta\,\tilde{t} + \tan^2\beta\left[1 - \exp\left(\frac{\widetilde{\eta}\cos\beta}{1+\alpha^2}\,\tilde{t}\right)\right]\right)$$

This function is shown for various β in Figure 1(a). We note that, as expected, increasing β leads to faster switching.

We want to find out whether, within some time $\tilde{\tau}$, $\hat{m}$ goes over the threshold $m_x > 0$ and gets closer to the final state than the initial state. If this does occur, then a spin pulse of duration τ will lead to successful

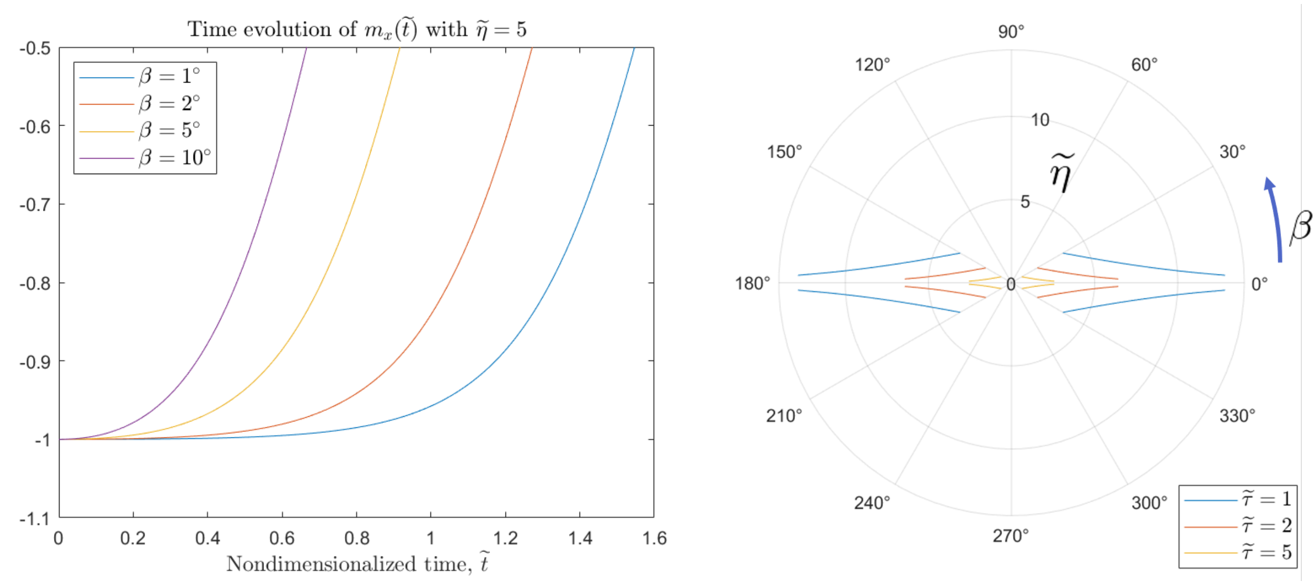

Figure 1: (a) the time evolution of the x-component of the magnetization from its initial condition $m_x = -1$. Note that higher β leads to faster switching. (b) the critical switching current $\widetilde{\eta}$ for a range of pulse durations $\widetilde{\tau}$ and in-plane spin polarization angles β. Note the "cloverleaf"-type dependence, also found in our comprehensive simulations. The model developed here is invalid for high β.

magnetization switching. This is how we find the critical switching thresholds. We note that in this result, η and t always appear together, so the relationship between the critical switching current and the critical pulse duration is a simple reciprocal one. (This is *not* the case with the full, real system, due to the effect of Gilbert damping – it only holds true in this simplified setup.)

As previously mentioned, the approximations that we have made only hold for small m_y and m_z, which implies m_x close to its initial condition of -1. Therefore, we cannot trace this function all the way to the threshold $m_x = 0$. Instead, we must extrapolate. We find, say, the $\widetilde{t}$ that leads to $m_x = -0.5$, and then double it to get the threshold $\widetilde{\tau}$. Simple linear extrapolation might not be too quantitatively accurate, especially in this system, but any method of extrapolation will produce qualitatively similar results as those shown below.

For ease of notation, let $g \equiv \widetilde{\eta}\widetilde{t}/(1+\alpha^2)$. We want to find the g such that $m_x = -m_T$, where m_T is the arbitrary threshold – we will then extrapolate from there.

$$m_T = \exp\left(\sin\beta \tan\beta \, g + \tan^2\beta \left[1 - e^{\cos\beta \, g}\right]\right)$$

$$\ln m_T - \tan^2\beta = \sin\beta \tan\beta \, g - \tan^2\beta \, e^{\cos\beta \, g}$$

$$\frac{\ln m_T}{\tan^2\beta} - 1 = \cos\beta \, g - e^{\cos\beta \, g}$$

$$g = \frac{1}{\cos\beta}\left(\left[\cot^2\beta \ln m_T - 1\right] - W_{-1}\left[-e^{1-\cot^2\beta \ln m_T}\right]\right)$$

where W is the real-valued negative branch of the Lambert W-function. Following our extrapolation technique, we can plug in $m_T = 0.5$ and then define the critical switching parameter $g_{\text{crit}} \equiv 2g$:

$$g_{\text{crit}} = -\frac{2}{\cos\beta}\left(1 + \cot^2\beta \ln(2) + W_{-1}\left[-e^{1+\cot^2\beta \ln(2)}\right]\right)$$

If we fix a $\widetilde{\tau}$, the critical switching current is then

$$\widetilde{\eta}_{\text{crit}} = -\frac{2(1+\alpha^2)}{\widetilde{\tau}\cos\beta}\left(1 + \cot^2\beta \ln(2) + W_{-1}\left[-e^{1+\cot^2\beta \ln(2)}\right]\right)$$

In Figure 1(b), we plot this relationship for a small range of β. Note that the "cloverleaf"-type dependence of the critical switching current found in the comprehensive simulations is recapitulated here, though the quantitative scaling of $\widetilde{\eta}$ is off.
